# eVITAL: A Preliminary Taxonomy and Electronic Toolkit of Health-Related Habits and Lifestyle

**DOI:** 10.1100/2012/379752

**Published:** 2012-04-01

**Authors:** Luis Salvador-Carulla, Carolyn Olson Walsh, Federico Alonso, Rafael Gómez, Carlos de Teresa, José Ricardo Cabo-Soler, Antonio Cano, Mencía Ruiz

**Affiliations:** ^1^Asociación Española para el Estudio Científico del Envejecimiento Saludable (AECES), Calle Infante Don Fernando 17, Málaga, 29200 Antequera, Spain; ^2^Asociación Científica PSICOST, Plaza de San Marcos 6, 11403 Jerez, Spain; ^3^Harvard Medical School, c/o Peabody Society, 260 Longwood Avenue, Boston, MA 02115, USA

## Abstract

*Objectives*. To create a preliminary taxonomy and related toolkit of health-related habits (HrH) following a person-centered approach with a focus on primary care. *Methods*. From 2003–2009, a working group (*n* = 6 physicians) defined the knowledge base, created a framing document, and selected evaluation tools using an iterative process. Multidisciplinary focus groups (*n* = 29 health professionals) revised the document and evaluation protocol and participated in a feasibility study and review of the model based on a demonstration study with 11 adult volunteers in Antequera, Spain. *Results*. The preliminary taxonomy contains 6 domains of HrH and 1 domain of additional health descriptors, 3 subdomains, 43 dimensions, and 141 subdimensions. The evaluation tool was completed by the 11 volunteers. The eVITAL toolkit contains history and examination items for 4 levels of engagement: self-assessment, basic primary care, extended primary care, and specialty care. There was positive feedback from the volunteers and experts, but concern about the length of the evaluation. *Conclusions*. We present the first taxonomy of HrH, which may aid the development of the new models of care such as the personal contextual factors of the International Classification of Functioning (ICF) and the positive and negative components of the multilevel person-centered integrative diagnosis model.

## 1. Introduction

Noncommunicable diseases cause 6 out of 10 deaths, and cardiovascular disease alone causes 31.5% of deaths in female and 26.8% in males [1]. Many of the leading causes of death have evidence-based modifiable risk factors [2–4], but this does not always translate to healthy behavior by individuals. Several studies have shown that risk of mortality or disease decreases stepwise based on the number of healthy habits practiced by an individual [5, 6]. In spite of the fact that major chronic diseases are caused by multiple risks, which when combined are associated with health outcomes, the science of multiple health behavior change and assessment is at an early stage, and factors that facilitate or impede success in investigative or clinical intervention in multiple behavior change are unknown [7].

 The developing field of longevity medicine takes a holistic view of health that calls for integrative evaluation of health-related habits (HrHs), both those that increase and decrease risk of disease and those related to general health and well-being, considering the endpoint of years lived without disability and taking into account a person-centered approach [8]. Taxonomies are particularly important in developing fields of study in that they standardize terminology and allow for common understanding of research results; recently proposed examples include the fields of adverse drug reactions [9] and patient-initiated medical errors [10]. In the current study, we present a preliminary taxonomy for HrH, as well as the Spanish version of the eVITAL toolkit for clinical evaluation of the lifestyle and related determinants of longevity of an individual.

## 2. Methods

Methods and ethics are described in detail elsewhere [11]. In short, the taxonomy and the related eVITAL toolkit were created using a nominal group technique involving a core group of 6 physicians with expertise in various aspects of longevity medicine and 29 health professionals, including physicians, nurses, and psychologists, in a series of four multidisciplinary focus groups. The model used in the creation of the taxonomy was adapted from the International Classification of Functioning, Disability and Health (ICF) [12] and other documents by the World Health Organization (WHO) [13–15], as well as the multilevel person-centered integrative diagnosis model [16], and the transtheoretical model of stages of change [17] and related model of multibehavior change [18]. According to the ICF a “domain” is “a practical and meaningful set of related physiological functions, anatomical structures, actions, tasks, or areas of life” [12]. “Dimensions” are the identifiable components of every domain. In some cases mutually exclusive domains could not be categorized and subdomains had to be defined (see below).

Entities were organized hierarchically into constructs, domains, subdomains, dimensions, subdimensions, and individual items, and codes were assigned using a hierarchical tree. In this conceptual model, health behaviors are part of HrH, complex behavioral patterns which are closely related to other determinants of health as well as to specific health conditions. HrH are in turn part of the health lifestyle, which is a key component of the “personal factors” defined in the ICF. These personal factors “are the particular background of an individual's life and living,” and these factors comprise, among others, “fitness, lifestyle, habits … overall behaviour pattern and character style, individual psychological assets and other characteristics, all or any of which may play a role in disability at any level” [12].

A demonstration study was performed with 11 adult volunteers who completed the evaluation package followed by an open-ended feedback questionnaire. The assessment package was then revised and computerized, the experts involved in the focus groups evaluated the feasibility of the online toolkit using the criteria of applicability, acceptability, and practicality [54], and responses were used to further refine eVITAL.

## 3. Results

### 3.1. Domains and Dimensions

The working group and experts revised 7 proposed domains (physical activity, diet, cognition, sleep, stress, psychosocial vitality, and risk behaviors) into the final 6 HrH domains by combining vitality and stress into a single domain combining physical activity and diet into one domain, and dividing “other risk behaviors” into the two domains of substance use and other risk habits (Table 2); the domains of cognition and sleep were unchanged. After discussion regarding the placement of sexuality within the hierarchy, it was decided that, while important for quality of life, sexuality does not meet all of the criteria for domains in terms of contributing to years lived without disability; it was therefore included as a subdimension within the vitality and stress domain. Despite the initial intention to only include evaluation of HrH, the working group decided that the clinical utility of the toolkit would be increased by including an assessment of other determinants and conditions of health specifically related to each basic HrH.

The panel suggested creating an overarching “health lifestyle profile,” with 6 subprofiles related to the 6 basic HrHs. A seventh domain, “Health descriptors,” includes generic descriptors of health related to longevity, such as social and medical determinants of health and current status of health.

The complete taxonomy developed through this process is shown in Table 3. The preliminary taxonomy includes 6 domains or classes (with diet/exercise further divided into three subdomains: generic, diet, and exercise), 43 dimensions or subclasses, and 141 subdimensions. Once the preliminary taxonomy was defined, codes were assigned to each entity and subentity following a hierarchical tree structure. Letters code the main branches or domains: cognition (c), vitality/stress (v), sleep (s), diet/exercise (de), substance use (s), and other risk habits (r). Each letter is followed by a number for the branches, or dimensions, except for the Prochaska stage of change which is coded within each domain by the letter “s” (see Table 3). The complete evaluation schema is shown in Table 4; the toolkit is available online at http://www.longevidad.org/.

Regarding cognition, the working group and expert panels included evaluation tools related to intellectual reserve or to a higher vulnerability to problems with memory or other higher cognitive functions. Tools were selected for the vitality and stress domain to evaluate psychological and social characteristics that are associated with longevity or an improved response to stress and illness. The group decided to include a biologic dimension to this domain due to the evidence linking stress to these components of allostatic load [55]. It was decided that, while diet and exercise have traditionally been considered separate domains, there is sufficient overlap in evaluation, clinical consequences, and intervention strategies that they should be combined. For example, both diet and exercise affect body mass index, which can be combined with activity level to form 16 metabolic types (Table 1). For substance use, the group considered 3 categories: substances that are always harmful such as nicotine and cocaine, those that can be health promoting in moderation such as wine and caffeine, and medication abuse, due to the potential harm done by misuse of all types of substances. For each substance, the following factors were considered: type/form, timing of use, amount consumed, degree of abuse, and related psychosocial and medical problems. The expert group separated substance use habits and non-substance-related risk habits due to differences in assessment, intervention, and evidence related to longevity. This final domain, “other risk habits,” is divided into treatment nonadherence and other risky behaviors; patient error related to treatment nonadherence is not further delineated within this preliminary taxonomy but has recently been described in detail [10].

### 3.2. Assessment Package

The evaluation is divided into four levels of increasing complexity, starting with basic self-assessment tools (Level 0) and progressing through assessments that can be completed in a basic primary care visit by a nonphysician provider (Level 1), in an extended primary care visit requiring physician expertise (Level 2), and in specialty care (Level 3). Within each level the evaluation is divided into two parts: anamnesis (items related to history) and medical exam.

The anamnesis includes 4 templates, 44 inventories, 22 rating scales, 5 sections (sleep, appetite, fatigue, obsessions, and hypochondriasis) of the semi structured interview “Standardized Polyvalent Psychiatric Interview” (SPPI) (also known by its Spanish acronym EPEP) [40], and 6 sub-classification systems (Table 4). We selected assessment instruments that were feasible at each assessment level according to level of complexity and need for trained expertise; when available, we prioritized items that had been standardized in Spain. When instruments were not available, the group designed inventories that should be standardized and validated at a later stage. In all, the full assessment package comprises 1078 items.

The medical exam includes physical exam findings (signs and measures) and laboratory tests. A series of standard indexes have been incorporated. The group designed adjusted indexes of cognitive reserve and body mass index that require future validation (Table 3).

**Figure 1:**
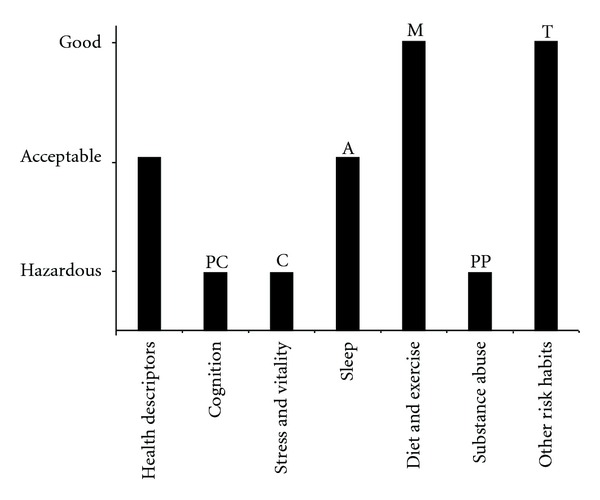
Sample lifestyle profile based on eVITAL toolkit. Prochaska stages: precontemplation (P), contemplation (C), preparation (PP), action (A), maintenance (M), termination (T).

The assessment package uses several possible methods of scoring the evaluation. In the simplest, after evaluating each domain, the rater gives a global impression score of the patient's profile for that domain in a 3-point Likert scale (good, acceptable, or needs improvement). These scores can be plotted for each of the domains in a health lifestyle profile and compared to the individual's stage of change for each domain to formulate a plan of care.  Figure 1 shows a sample assessment. This type of assessment and its related lifestyle profile can be extended to the dimensions, subdimensions, and types.

### 3.3. Demonstration Study

Characteristics of the 11 adult volunteers were as follows: mean age 57.45 years (range 43–64), 9 male, marital status: 9 married/1 widow/1 single, 6 with university degrees, all upper-middle income. Problems in HrH were identified in all volunteers: 8 individuals had problems with sleep, 8 with diet, 5 with exercise, 5 with substance use, 2 with other risk habits, and 2 with vitality/stress. Cognitive habits were good or acceptable in all individuals. 10 individuals fulfilled at least one diagnosis from the International Classification of Diseases (ICD-10) [56] in spite of perceiving themselves as “healthy” (Table 2).

After completing the toolkit, 9/11 gave an overall favorable review and 11/11 reported favorable interactions with the professionals administering the evaluation. While there were no specific recommendations for changes from the volunteers, 7/11 reported that the evaluation was quite long.

### 3.4. Feasibility Study

Upon reviewing the results of the demonstration study, the working group and focus groups revised the basic organization of the assessment package. Then a feasibility questionnaire was sent to the 29 experts involved in the focus groups; 15 responses were received suggesting changes while 14 experts judged the previous package as adequate and provided no further comments. Comments about applicability of the survey were generally positive. In terms of acceptability, there was some concern about generalizability to populations with lower education level and socioeconomic status, as well as whether patients would be able to complete the forms without assistance. Regarding practicality, there was concern about the time required of the clinician, as well as the difficulty of managing all of the data gathered. As the ultimate goal is to integrate eVITAL into use in the primary care system, comments from primary care practitioners, such as the following, were particularly important: “The survey seems too ambitious and impractical for primary care … a tool that you cannot use due to lack of resources (above all, time) loses its practical validity.”

### 3.5. Development of the Toolkit

These comments were taken into account in developing the electronic toolkit eVITAL. The open access preliminary version of the toolkit is available at http://www.longevidad.org/inicio.

## 4. Discussion

Although there is an increasing interest in the comprehensive assessment of HrHs and their relationship to longevity [57], this study presents the first attempt at classifying HrHs to date using the longevity model with the endpoint of years lived without disability. The ICF indicates the relevance of HrH and lifestyle as main components of the “personal contextual factors,” but these factors have not been defined or coded to date [58].

The transtheoretical model of stages of change [17] with the related multibehavioral assessment [18] is the main integrative approach to HrH. Despite the limited evidence regarding the effectiveness of stage-based interventions as a basis for behavior change or for facilitating stage progression [59], multiple behavioral assessment provides a composite index of overall behavior change and includes overarching outcome measures such as quality of life, related biometrics, and cost [60]. For example, a composite index for evaluating change in physical activity and diet showed that interventions focused only on exercise achieved a larger amount of behavior change than an intervention combining both physical activity and nutrition [60].

The eVITAL expert panel opted for a global impression rating of every major HrH and the graphical representation of the resulting lifestyle (health profile), instead of using composite indexes. Similar global ratings have been shown to be practical both in routine clinical practice [61] and in eHealth tools [62].

Unexpectedly given the income and the education level of the volunteers in our pilot sample, we found numerous HrHs in the “needs improvement” category, along with illnesses both related and unrelated to HrH. We diagnosed one case each of prostate cancer and Sjögren's syndrome, as well as a high proportion of sleep, diet, and exercise problems. This pilot may indicate the relevance of designing both population- and primary care-based epidemiological studies of health lifestyles which include all basic habits and related conditions, as opposed to focusing specifically on targets such as nutrition or exercise.

### 4.1. Study Strengths

This study is unique in its integrative approach to the evaluation of HrH and the focus on the middle-aged adult population. It begins to address the barriers to health promotion in the primary care setting recently identified in Spain [63] by providing an innovative approach to the assessment of individuals. This preliminary taxonomy fills an existing gap in the assessment of HrH. The eVITAL toolkit is freely available online for use in clinical and research settings, with the hope that this and other groups will continue to gather information on its utility and contribute to further refinement.

### 4.2. Study Limitations

The taxonomy has received adequate consensus, and the related tools included in eVITAL are those deemed by group to be most useful in the development of an integrated understanding of the HrH and lifestyle of an individual in the Spanish cultural context. However, the clinical utility of the toolkit as a whole will have to be validated in the future. The current, computerized version of eVITAL has not undergone the type of demonstration study reported here for the earlier assessment package prototype; this remains to be performed prior to widespread integration into clinical practice. The greatest limitation of the toolkit at this time is the concern raised about the feasibility of widespread use of eVITAL, most notably in populations with lower education level and socioeconomic status and in the primary care system. While it is important to gather enough information to develop a complete understanding of a patient's health lifestyle profile, the system we propose must be feasible within the existing health care system. eVITAL will continue to be adjusted to work toward this goal.

## 5. Conclusion

To our best knowledge this is the first toolkit of lifestyle and health-related habits based on a formal taxonomy of HrH. This taxonomy may improve the assessment of lifestyle in health sciences, enhance the development of a classification of HrH and personal factors in the context of the WHO family of classifications, and develop this construct in new models of care such as person-centered medicine and diagnosis [16, 64].

## Figures and Tables

**Table 1:** Metabolic classification based on body mass index and physical activity (eVITAL).

Body mass index (BMI)	Physical activity
(a) Underweight (BMI <18.5)	(i) Sedentary
(b) Normal weight (BMI 18.5–25)	(ii) Daily activity, no purposeful exercise
(c) Overweight (BMI 25–30)	(iii) Regular exercise
(d) Obese (BMI >30)	(iv) Very active

**Table 2:** eVITAL lifestyle profile related to health habits and the Prochaska stage of change in 11 volunteers.

Subject	Cognition	Vitality and stress	Sleep	Diet and exercise		Substance use	Other risk habits	Main health conditions (ICD-10)
				Ex.	Diet			
1	↑	↑	↓	↓M	↓P	↑	↑	Insomnia, sleep apnea, overweight, high cholesterol
2	↑	↑	—	—C	—M	—M	↑	High cholesterol
3	↑	↓	↓	↓P	↓PR	↑	↑	Sleep apnea (mild), osteoporosis, Sjögren's syndrome
4	↑	↑	↓	↓C	↓P	↓C	↓C	Sleep apnea, overweight/sedentariness, nicotine abuse, hypertension
5	↑	↑	↓	—M	↓P	↓P	↑	Sleep apnea (mild), overweight
6	↑	↑	↓	—M	↓M	↑	↑	Sleep apnea (mild)
7	↑	↑	↑	—M	—M	↑	↑	Anxiety
8	↑	↓	↓	↓P	↓P	↑	↑	Anxiety, insomnia, overweight/sedentariness, high cholesterol
9	↑	↑	↓	↓R	↓R	↓P	↑	Sleep apnea (mild), sedentariness, nicotine abuse, diabetes (type 2)
10	—	↑	↓	—C	—P	↑	—P	Sleep apnea, prostate cancer
11	—	↑	↑	—C	↓C	↓C	↓A	Anxiety, overweight, nicotine abuse

eVITAL Health stage: good (↑), acceptable (**—**), needs improvement (↓), the Prochaska stage of change (in domains where this is highly relevant and the global impression rating is acceptable or needs improvement): precontemplation (P), contemplation (C), preparation (PP), action (A), maintenance (M), termination (T), relapse (R). Subjects were 11 adult volunteers in Antequera, Spain, who reported themselves to be healthy. Assessment occurred in 2008.

**Table 3:** Classification system of health-related behaviors (7 domains, 3 subdomains, 43 dimensions, and 141 subdimensions).

h. Domain: health descriptors		

Dimensions (5)	Subdimensions (14)	

h.1. Sociodemographics	h.1.1. Personal information	
	h.1.2. Information about partner	
	h.1.3. Work	
	h.1.4. Ethnicity and culture	

h.2. Family history	h.2.1. Ancestors and siblings	
	H.2.1.1. Longevity	
	H.2.1.2. Medical history	
	h.2.2. Descendants	
	H.2.2.1. Longevity	
	H.2.2.2. Medical history	

h.3. Developmental history	h.3.1. Infancy	
	h.3.2. Childhood	
	H.3.2.1. Generic	
	H.3.2.2. Laterality	

h.4. Past medical history	h.4.1. Diseases/health conditions	
	H.4.1.1. Generic	
	H.4.1.2. Related symptoms	
	h.4.2. Surgical history	
	h.4.3. History of trauma or injury	

h.5. Medical treatments	h.5.1. Medications	
	H.5.1.1. Generic	
	H.5.1.2. Immunization history	
	H.5.1.3. Hormonal therapy	
	h.5.2. Alternative therapies	
	h.5.3. Other medical care within past year	

c. Domain: cognition		

Dimensions (7)	Subdimensions (19)	

c.1. Cognitive reserve (CR)	c.1.1. Education level	
	c.1.2. Current intellectual activity	
	c.1.3. Current rating	
	C.1.3.1. Global cognitive reserve (gCR)	
	C.1.3.2. Global cognitive performance (gCP)	
	C.1.3.3. Cognitive reserve adjusted for risk (CRar)	

c.2. Attention	c.2.1. Attention generic	

c.3. Memory	c.3.1. Verbal semantic memory	
	C.3.1.1. Unprompted	
	C.3.1.2. With prompts	
	c.3.2. Visual memory	
	C.3.2.1. Items correct	
	C.3.2.2. Errors	

c.4. Learning	c.4.1. Learning curve	
	c.4.2. Consolidation	
	c.4.3. Primacy	
	c.4.4. Recency	
	c.4.5. Errors made during learning	
	c.4.6. Improvement with repeated exposure	

c.5. Other intellectual functions	c.5.1. Visuospatial ability	
	c.5.2. Motor speed	
	c.5.3. Perception	
	c.5.4. Executive function	

c.6. Global cognitive decline	c.6.1. Global assessment	
	c.6.2. Spatial/temporal orientation	

*c.s* - *Stage of change *	c.s.1. Observed	

v. Domain: vitality and stress		

Dimensions (7)	Subdimensions (28)	

v.1. Vitality	v.1.1. Happiness	
	v.1.2. Optimism	
	v.1.3. Vital state	
	v.1.4. Sense of purpose	
	v.1.5. Sense of control (mastery)	
	v.1.6. Responsibility	
	v.1.7. Sexuality	

v.2. Social reserve	v.2.1. Positive relationships	
	v.2.2. Isolation	
	v.2.3. Social support	

v.3. Psychosomatic reserve	v.3.1. Amiability	
	v.2.2. Self-restraint	
	v.3.3. Type behavioral pattern (A, B)	
	v.3.4. Related health conditions	
	v.3.4.1. Anxiety	
	v.3.4.2. Depression	
	v.3.4.3. Rumination/obsessive tendencies	
	v.3.4.4. Preoccupation with health	

v.4. Major life events	v.4.1. Number	
	v.4.2. Impact	

v.5. Psychological resistance to stress	v.5.1. Emotional stability	
	v.5.2. Extraversion	
	v.5.3. Anxiety (trait)	
	v.5.4. Distress	
	v.5.4.1. Internalized distress	
	v.5.4.2. Psychological distress	
	v.5.5. Emotional State	

v.6. Basic allostatic load	v.6.1. Blood pressure	
	v.6.2. Waist-to-hip ratio	
	v.6.3. HDL	
	v.6.4. Total cholesterol/HDL	
	v.6.5. Glycated hemoglobin	
	v.6.6. C-reactive protein	

*v.s - Stage of change*	v.s.1. Observed	

s. Domain: sleep		

Dimensions (5)	Subdimensions (12)	

s.1. Sleep habits and quality	s.1.1. Sleep schedule	
	s.1.2. Sleep quality	
	s.1.3. Other sleep-related behaviors	
	s.1.4. Ingestions related to sleep	

s.2. Insomnia	s.2.1. Generic	
	s.2.2. Pattern	
	S.2.2.1. Onset insomnia	
	S.2.2.2. Middle insomnia	
	S.2.2.3. Late insomnia	

s.3. Hypersomnia	s.3.1. Somnolence	
	s.3.2. Nonrestorative sleep	

s.4. Other sleep problems (related health conditions)	s.4.1. Sleep apnea	
	s.4.2. Restless legs	
	s.4.3. Other sleep problems	

*s.s - Stage of change*	s.s.1. Observed	

de. Domain: diet and exercise de. Subdomains (3) (i) deg—GENERIC (ii) Specific (a) d—Diet (b) e—Exercise		

Dimensions deg. (3)	Subdimensions deg. (8)	

deg.1. Body composition (BC) (body mass index (BMI))	deg.1.1. BMI (generic)	
	deg.1.2. BMIa (adjusted)	
	deg.1.3. BMIar (adjusted for risk)	

deg.2. BC components (fat and lean body mass)	deg.2.1. Estimated BCC (anthropometrics formulas and standardized calculation tables)	
	deg.2.2. Indirect BCC (impedance testing) BC	
	deg.2.3. Direct BCC (DEXA)	

deg.3. Metabolic type (see Tables 3 and 4)	deg.3.1. Classification MT: 16 types—5 recommendations levels	
	deg.3.2. METs	
	deg.3.2.1. Estimated METs (BMI + daily activity classification).	
	deg.3.2.2. Indirect METs (interview-oriented food + CPA tables).	
	deg.3.2.3. Direct METs (nutritional calculator and ergometry).	

Specific^(1)^ d + e (4 + 4 = 8)	d. Diet—subdimension (12)	e. Exercise—subdimension (14)

Experiences	d.1. Experiences of appetite and weight change	e.1. Fatigue during activity
	d.1.1. Change in appetite	e.1.1. Intensity
	d.1.2. Change in weight	e.1.2. Frequency
		e.1.3. Fatigue during leisure activities
		e.1.4. Degree of interference with activity
		e.1.5. Duration

Habits	d.2. Diet habits	e.2. Activity habits
	d.2.1. Schedule	e.2.1. Self-reported activity level
	d.2.2. Perception of diet	e.2.2. Degree of sedentariness
	d.2.3. Basic eating habits	e.2.3. Exercise habits
	d.2.4. Type of diet	e.2.4. Work-related physical activity
	d.2.5. Quality of diet	

Health-related conditions—medical factors	d.3. Health-related conditions (medical dietary factors)	e.3. Health-related conditions (medical activity factors)
	d.3.1. Digestive problems	e.3.1. Musculoskeletal
	d.3.2. Food intolerance	e.3.2. Cardiovascular
	d.3.3. Dental problems	e.3.3. Respiratory

*de.s. Stage of change: habits*	d.s. Stage of change—diet	e.s. Stage of change—exercise
	d.s.1. Observed	e.s.1. Observed
	d.s.2. Subjective	e.s.2. Subjective

u. Domain: substance use		

Dimensions (6)	Subdimensions (30)	

u.1. Medications	u.1.1. Type	
	u.1.2. Initiation	
	u.1.3. Intensity of consumption	
	u.1.4. Level of abuse	
	u.1.5. Psychosocial and medical problems	

u.2. Caffeine	u.2.1. Type	
	u.2.2. Initiation	
	u.2.3. Intensity of consumption	
	u.2.4. Level of abuse	
	u.2.5. Psychosocial and medical problems	

u.3. Nicotine	u.3.1. Type	
	u.3.2. Initiation	
	u.3.3. Intensity of consumption	
	u.3.4. Level of abuse	
	u.3.5. Psychosocial and medical problems	

u.4. Alcohol	u.4.1. Type	
	u.4.2. Initiation	
	u.4.3. Intensity of consumption	
	u.4.4. Level of abuse	
	u.4.5. Psychosocial and medical problems	

u.5. Illicit Drugs	u.5.1. Type	
	u.5.2. Initiation	
	u.5.3. Intensity of consumption	
	u.5.4. Level of abuse	
	u.5.5. Psychosocial and medical problems	

*u.s. Stage Of Change*	u.s.1. Medications	
	u.s.1.1. Observed	
	u.s.1.2. Subjective	
	u.s.2. Caffeine	
	u.s.2.1. Observed	
	u.s.2.2. Subjective	
	u.s.3. Nicotine	
	u.s.3.1. Observed	
	u.s.3.2. Subjective	
	u.s.4. Alcohol	
	u.s.4.1. Observed	
	u.s.4.2. Subjective	
	u.s.5. Illicit drugs	
	u.s.5.2. Observed	
	u.s.5.1. Subjective	

r. Domain: other health risk habits		

Dimensions (2)	Subdimensions (4)	

r.1. Nonadherence to treatment	r.1.1. Generic	
	r.1.s Stage of change	
	r.1.s.1. Observed	
	r.1.s.2. Subjective	

r.2. Other risk behaviors	r.2.1. Type	
	r.2.2.1. Risky sexual behavior	
	r.2.2.2. Dangerous sports	
	r.2.2.3. Gambling	
	r.2.2.4. Dangerous driving	
	r.2.2.5. Other risk behaviors (e.g., sun exposure)	
	r.2.s Stage of change	
	r.2.s.1. Observed	
	r.2.s.2. Subjective	

^(1)^Specific subdimensions of diet/exercise are listed one level down from where they are in other domains.

**Table 4:** Evaluation of items included in the eVITAL toolkit^(1)^.

1. Domain: health descriptors		

Evaluation	Instruments	
History—Level 0	Sociodemographic inventory	
History—Level 1	Family medical history	
	Personal medical history	
	Medication, hormone use, and alternative treatments	
	Immunization history	
History—Level 2		
History—Level 3		
Examination—Level 0		
Examination—Level 1		
Examination—Level 2		
Examination—Level 2		

2. Domain: cognition		

Evaluation	Instruments	
History—Level 0	Screening for cognitive problems	
History—Level 1	Cognitive reserve (CR)	
History—Level 2	(i) Cognitive risk factors	
	(ii) Cognitive reserve adjusted for cognitive risk (CRar)	
	(iii) Stage of change, objective [17]	
History—Level 3	Global cognitive reserve (gCR)	
Examination—Level 0		
Examination—Level 1		
Examination—Level 2	7-minute test [19, 20]	
Examination—Level 3	(i) Luria's test [21]	
	(ii) Benton's visual recognition test [22]	
	(iii) Trail making test-A (TMT-A) [23]	
	(iv) Finger electronic tapping test (FETT) [24]	

3. Domain: vitalty and stress		

Evaluation	Instruments	
History—Level 0	Screening for emotional state	
History—Level 1	(i) Optimism-LOT-R: life orientation test-revised [25]	
	(ii) Emotional stress—Questions 5–9, SF-36: social functioning-36 question scale [26, 27]/MHI-5.	
	(iii) Social isolation—STAKES [28]	
	(iv) Social support—Oslo [29]	
	(v) Anxiety/depression—HAD: hospital anxiety and depression scale [30]	
History—Level 2	(i) NEO Pi-R: revised NEO personality inventory [31]	
	(ii) Psychological well-being [32, 33]	
	(iii) Sexuality quality of life: sexuality subscales [34, 35]	
	(iv) Stress—HAD [30, 36]	
	(v) Stage of change, objective [17]	
History—Level 3	(i) Social readjustment rating scale [37]	
	(ii) Type A personality—ERCTA: escala retiro de patrón de conducta tipo A (scale of Type A behavior pattern) [38]	
	(iii) Aggression subscale—ZKPQ: Zuckerman-Kuhlman's personality questionnaire [39]	
	(iv) Obsessions—SPPI: standardized polyvalent psychiatric interview [40]	
	(v) Hypochondriasis—SPPI [40]	
	(vi) Gender-specific medical evaluation, including breast cancer risk assessment [41]	
Examination—Level 0		
Examination—Level 1		
Examination—Level 2	Basic allostatic load: blood pressure, waist-to-hip ratio, total/HDL cholesterol, HDL, glycated hemoglobin, CRP	
Examination—Level 3	Expanded allostatic load: add DHEA-S and urinary cortisol	

4. Domain: sleep		

Evaluation	Instruments	
History—Level 0	Screening for insomnia and hypersomnia	
History—Level 1	(i) Insomnia—SPPI [40]	
	(ii) Somnolence—Epworth sleepiness scale [42–44]	
	(iii) Specific sleep-related symptoms	
	(iv) Sleep habits	
History—Level 2	Stage of change, objective [17]	
History—Level 3		
Examination—Level 0		
Examination—Level 1		
Examination—Level 2		
Examination—Level 3	Polysomnography	

5. Domain: diet and exercise		

Evaluation	Instruments	
	Instruments DIET	Instruments EXERCISE
History—Level 0	Screening for change in appetite (SSPI)	Activity level
History—Level 1	Eating habits	Exercise habits
	Table of mealtimes	Exercise readiness—Par-Q: physical activity readiness questionnaire [45]
	Appetite-SPPI [40]	Fatigue-adapted SPPI [40]
	Subclassification BMI	Estimated METs
	Metabolic classification (combines weight status and activity level) (16 levels)	
	Classification as recommendations: 5 levels	
	Readiness to change—diet and exercise—subjective	Readiness to change—diet and exercise—objective
History—Level 2	CFCA-food frequency questionnaire [46]	
	Digestive symptoms, food intolerance, dental problems	Musculoskeletal physical barriers to exercise
	Readiness to change—diet and exercise—objective	Readiness to change—diet and exercise—objective
History—Level 3		
Examination—Level 0	(i) Waist circumference	
	(ii) Reported body mass index (BMI) from reported weight and height	
Examination—Level 1	(i) Body composition—subclassification BMI	
	(ii) Waist-to-hip ratio	
	(iii) BMI adjusted for waist circumference [47], (BMIa)	
Examination—Level 2	(i) Basic lab work: total/HDL cholesterol, HDL, glycated hemoglobin, C-reactive protein, microalbumin, glucose, insulin	
	(ii) Blood pressure, heart rate.	
	(iii) Adjusted BMI (for adult weight gain, triglyceride/HDL, blood pressure, fasting glucose, and presence or absence of sleep apnea and osteoarthritis (BMIar))	
	(iv) Body composition (triceps skin fold), estimated muscle mass (arm circumference)	
	(v) Insulin resistance-HOMA-IR [48]	
Examination—Level 3	3-day food record analyzed by nutritionist	Indirect METs calculated through compendium of physical activities—CPHA [49]
		Hand strength via dynamometry
	(i) Body composition via BIA or DEXA	(i) Somatometry
	(ii) Nutritional calculator	(ii) Ergometry
		(iii) Direct METs measurement

6. Domain: substance abuse		

Evaluation	Instruments	
History—Level 0	Screening for substance abuse	
History—Level 1	Inventory: use and abuse of prescription medications, use of caffeine, nicotine, alcohol, and illicit drugs	
	Adapted CAGE questionnaire for each substance [50, 51]	
	Stage of change for each substance, subjective	
History—Level 2	(i) Stage of change for each substance, objective	
	(ii) For smokers: Fagerström's test of nicotine dependence [52, 53]	
History—Level 3		
Examination—Level 0		
Examination—Level 1		
Examination—Level 2		
Examination—Level 3	Nicotine—CO-oximetry	
	Alcohol—GGT, MCV	
	Drug levels in urine/blood/hair tests	

7. Domain: other risk habits		

Evaluation	Instruments	
History—Level 0		
History—Level 1	(i) Medication adherence.	
	(ii) Inventory of risk behaviors: risky sexual behavior, dangerous sports, gambling, dangerous driving, potentially dangerous travel, internet/technology addiction.	
	(iii) Readiness to change—subjective	
History—Level 2	Readiness to change—objective	
History—Level 3		
Examination—Level 0		
Examination—Level 1		
Examination—Level 2		
Examination—Level 3		

^(1)^All tests are fully described at the eVITAL webpage (http://www.longevidad.org/). References can be downloaded at http://www.longevidad.org/descargas/011anexo_bibliografia_final.pdf.

BIA: bioelectrical impedance analysis; CO: carbon monoxide; CRP: C-reactive protein; DEXA: dual-energy X-ray absorptiometry; GGT: gamma-glutamyl transpeptidase; HDL: high-density lipoprotein; HOMA: homeostatic model assessment; MCV: mean corpuscular volume; MHI: mental health index.

## Abbreviations

BMI: Body mass index
ICD: International Classification of Diseases
ICF: International Classification of Functioning, Disability and Health
HrH: Health-related habits
SPPI: Standardized Polyvalent Psychiatric Interview
WHO: World Health Organization.
